# Analysis of microbial communities in the oil reservoir subjected to CO_2_-flooding by using functional genes as molecular biomarkers for microbial CO_2_ sequestration

**DOI:** 10.3389/fmicb.2015.00236

**Published:** 2015-03-31

**Authors:** Jin-Feng Liu, Xiao-Bo Sun, Guang-Chao Yang, Serge M. Mbadinga, Ji-Dong Gu, Bo-Zhong Mu

**Affiliations:** ^1^State Key Laboratory of Bioreactor Engineering and Institute of Applied Chemistry, East China University of Science and TechnologyShanghai, China; ^2^School of Biological Sciences, University of Hong KongHong Kong, China

**Keywords:** CO_2_ fixation, bioconversion, methane, functional genes, oil reservoir, microbial communities

## Abstract

Sequestration of CO_2_ in oil reservoirs is considered to be one of the feasible options for mitigating atmospheric CO_2_ building up and also for the *in situ* potential bioconversion of stored CO_2_ to methane. However, the information on these functional microbial communities and the impact of CO_2_ storage on them is hardly available. In this paper a comprehensive molecular survey was performed on microbial communities in production water samples from oil reservoirs experienced CO_2_-flooding by analysis of functional genes involved in the process, including *cbbM*, *cbbL*, *fthfs*, [FeFe]-hydrogenase, and *mcrA*. As a comparison, these functional genes in the production water samples from oil reservoir only experienced water-flooding in areas of the same oil bearing bed were also analyzed. It showed that these functional genes were all of rich diversity in these samples, and the functional microbial communities and their diversity were strongly affected by a long-term exposure to injected CO_2_. More interestingly, microorganisms affiliated with members of the genera *Methanothemobacter*, *Acetobacterium*, and *Halothiobacillus* as well as hydrogen producers in CO_2_ injected area either increased or remained unchanged in relative abundance compared to that in water-flooded area, which implied that these microorganisms could adapt to CO_2_ injection and, if so, demonstrated the potential for microbial fixation and conversion of CO_2_ into methane in subsurface oil reservoirs.

## Introduction

Storage of CO_2_ in deep geological formations, such as oil reservoirs, is one of the feasible measures to reducing CO_2_ emissions into the atmosphere. Understanding the fate of CO_2_ in the subsurface environment is of great scientific interest and significance, and has received increasing attention for more information to assess the feasibility. Due to the fact that abundant microorganisms inhabit in these formations, microbial fixation and conversion of the sequestered CO_2_ into CH_4_ are becoming an area of active research and development.

After CO_2_ injection, characteristics of the formation water may be changed by CO_2_ dissolution, including pH, the availability of inorganic and organic components in the brine, microbial attachment and biofilm formation as well as the microbial activities at *in situ* oil reservoirs. Generally, as CO_2_ is also a potential source of carbon of chemolithoautotrophic microorganisms such as methanogens, the injected CO_2_ may activate these microorganisms and notably influence the microbial structure and their activity *in situ*. Studies have been performed on the physical and chemical changes in the CO_2_ storage sites. The first on-shore CO_2_ storage site in Europe was established, and the effects and feasibility of CO_2_ injection and storage in a 650m deep saline aquifer was examined (Wandrey et al., 2011). The potential of microbial conversion of CO_2_ into CH_4_ by hydrogenotrophic methanogens isolated from oil reservoirs has been evaluated based on laboratory experiments by Sugai et al. (2012). As to the microbial involvement, six autotrophic CO_2_ fixation pathways were documented, of which the Calvin–Benson–Bassham (CBB) cycle plays an important role in autotrophic CO_2_ fixation (Berg, 2011). The CBB biochemical process was reported to occur in *Proteobacteria*, including some members of *Firmicutes*, *Actinobacteria*, and *Chloroflexi* as well as in plants, algae, and cyanobacteria (Ivanovsky et al., 1999; Zakharchuk et al., 2003; Berg et al., 2005; Caldwell et al., 2007; Lee et al., 2009). Another important pathway of CO_2_ fixation is the reductive acetyl-CoA pathway that has documented to occur in acetogenic prokaryotes, ammonium-oxidizing *Planctomycetes* (Strous et al., 2006), sulfidogenic bacteria (Schauder et al., 1988), and autotrophic archaea affiliated with the order *Archaeoglobales* (Vorholt et al., 1995, 1997). This pathway is also utilized by acetogenic prokaryotes for energy conservation (Ragsdale and Pierce, 2008; Thauer et al., 2008; Biegel and Muller, 2010).

**Figure 1:**
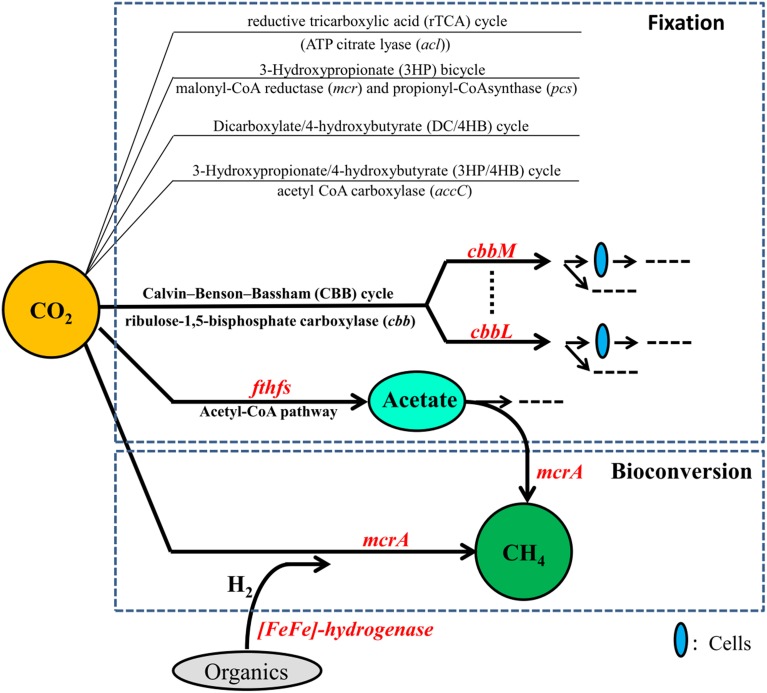
**Genes and pathways for CO_2_ fixation and bioconversion into CH_4_**.

Petroleum reservoirs are known to harbor diverse microorganisms including bacteria such as *Proteobacteria*, *Firmicutes*, *Actinobacteria*, and *Chloroflexi* and archaea such as methanogens and *Archaeoglobales* mentioned above (Magot et al., 2000; Li et al., 2010, 2011; Wang et al., 2011; Mbadinga et al., 2012) and they are expected to fix and/or convert CO_2_ into CH_4_ more effectively. To investigate whether oil reservoirs have the potential of CO_2_ biofixation and bioconversion of CO_2_ into CH_4_, and to have a better knowledge on microorganisms involved in this process and the impact of long-term CO_2_ exposure on them, studies from a viewpoint of functional genes are necessary. Functional genes involved in CO_2_ fixation and conversion into CH_4_ have been shown to be valuable functional biomarkers for detecting the microbial communities both in environments and enrichment cultures. The genes *cbbL* and *cbbM* respectively encoding the key enzymes ribulose 1,5-bisphosphate carboxylase/oxygenase (RubisCO) form I and II of the CBB cycle for CO_2_ fixation have been used to study microbial communities from hydrothermal vents of the Logatchev field (Hugler et al., 2010). The gene *fthfs* encoding formyltetrahydrofolate synthetase, a key enzyme in the reductive acetyl-CoA pathway, has been used to investigate the diversity of homoacetogenic bacteria in thermophilic and mesophilic anaerobic sludge (Ryan et al., 2008). Methyl-Coenzyme M reductase (*mcr*) is vital for CH_4_ formation, and the α-subunit of MCR (*mcrA* gene) is commonly used in the detection of specific groups of methanogenic communities (Juottonen et al., 2006). In addition, H_2_ should be supplied in the process of CO_2_ conversion into CH_4._ H_2_ can be produced by H_2_-producing prokaryotes which are polyphyletic. [Fe-Fe]-hydrogenases are known to catalyze H_2_ production in fermentative microorganisms, and thus gene encoding for *[Fe-Fe]*-hydrogenases represent a good marker gene for the detection of H_2_-producing anaerobes (Schmidt et al., 2010). These valuable functional biomarkers involved in CO_2_ fixation and conversion into CH_4_ are shown in Figure 1.

The objectives of this study were to evaluate the potential of *in situ* microbial fixation and conversion of CO_2_ into CH_4_ in subsurface oil reservoir through analysis of functional genes (*cbbM*, *cbbL*, *fthfs*, gene encoding by [FeFe]-hydrogenase, and *mcrA*) by: (1) characterization of the functional microbial communities involved in this process in the production waters from CO_2_-flooded and water-flooded areas, respectively, of the same high-temperature oil-bearing bed in Daqing Oilfield; and (2) Analysis of the impact of long-term exposure of CO_2_ on these functional microbial communities.

## Materials and methods

### Sampling site and production water samples

Production water samples were collected from production wells (designated as C and W) in YSL block of Daqing oilfield, China. At that time, the water cut of fluid from C and W production wells were 15 and 11%, respectively. The CO_2_ injected had been produced from the sampling well about 1 year before, and the ratio of gas (CO_2_) to oil was between 22.8 and 145 m^3^/m^3^ in production wells. The distance between injection well and the sampling production well is about 250 m. These wells produced oil from the same oil-bearing bed but C is located in the area subjected to CO_2_ flooding since 2007, whereas W by water-flooding only. To date, about 100,000m^3^ of liquid CO_2_ have been injected into the oil-bearing bed with an average injection rate of 10 m^3^/d per injection well in a manner of CO_2_-H_2_O alternate injection for Enhancement of Oil Recovery (EOR). These samples were taken through sampling valves located at the wellhead (average temperature 45°C) and put into 5L sterile bottles, respectively, to fullness, and then capped and sealed to maintain anoxic conditions. The bottles were kept at 4°C before further treatment. The *in situ* temperature and pressure of the target oil-bearing bed with a depth of about 2000m were about 90°C and 19 MPa, respectively. The average density of oil in this oil-bearing bed is 0.8581 g/cm^3^ and the information of the production water is listed in Table 1.

**Table 1:** **Characteristics of the production water samples**.

**Parameter**	**C**	**W**
pH	6.4	6.0
Salinity (mg L^−1^)	3897	3920
Cl^−^ (mg L^−1^)	1947	1872
SO^2-^_4_ (mg L^−1^)	667	808
PO^3-^_4_ (mg L^−1^)	nd	nd
NO^−^_3_ (mg L^−1^)	nd	nd
Na^+^ (mg L^−1^)	1110	1115
NH^+^_4_ (mg L^−1^)	24.6	25.8
K^+^ (mg L^−1^)	6.8	6.9
Ca^2+^ (mg L^−1^)	131.5	83.1
Mg^2+^ (mg L^−1^)	10.0	8.9
Mn^2+^ (mg L^−1^)	nd	nd
Formate (mg L^−1^)	nd	nd
Acetate (mg L^−1^)	109.1	7.7
Propionate (mg L^−1^)	nd	nd
Isobutyrate (mg L^−1^)	2.5	2.9
Butyrate (mg L^−1^)	nd	nd
⊥tomrule

*pH, anion, cation were analyzed by pH meter, ion chromatography, and ICP-AES (Inductively Coupled Plasma-Atomic Emission Spectrometry), respectively; Volatile fatty acids were determined by GC-MS after butanol esterification; nd, not detectable*.

### DNA extraction

DNA was extracted from the oil/water sample according to the method previously described by Wang et al. (2012). Briefly, the water phase was separated from the oil/water mixture by heating the samples to 50°C and by phase separation in sterilized separatory funnels. The microbial biomass in the water fraction was concentrated onto membrane filter (0.22-μ m-pore-size). Total genomic DNA of samples was extracted from 2.0 L of production water samples using AxyPrep™ Bacterial Genomic DNA Miniprep Kit (Axygen Biosciences, Inc., CA, USA) according to the manufacturer's DNA Miniprep spin protocol after concentration onto membrane filters. The genomic DNAs obtained were purified with a DNA purification kit (U-gene, China) according to the manufacturer's instructions. The extracted DNAs were stored at −20°C until PCR amplification of different functional genes (Wang et al., 2012).

### PCR amplifications

Amplifications of the *cbbL* gene fragment (771 bp) and the *cbbM* gene fragment (328 bp) were carried out under the conditions described by Campbell and Cary (2004). For amplification of a portion (1102 bp) of the *fthfs* gene, the PCR conditions used were those described previously by Leaphart and Lovell (2001). For amplification of a fragment (620 bp) of [Fe-Fe]-hydrogenase-encoding gene, the PCR primer set HydH1f/HydH3r was applied using the conditions described by Schmidt et al. (2010). A fragment (470 bp) of the *mcrA* genes was amplified using the primer set MLf/MLr (Luton et al., 2002) with the conditions as reported previously (Galand et al., 2005). Functional genes fragments were all amplified in five parallel PCR reactions in a Peltier thermal cycler (Bio-Rad, USA), which were subsequently pooled for cloning and construction of genes libraries.

### Construction of functional genes clone libraries

The amplified and pooled PCR products were gel-purified using the Gel Extraction Kit (U-gene, China) and then cloned into *Escherichia coli* using a pMD19^®^-T simple vector kit (Takara, Japan) following the instructions of the manufacturer. For each gene clone library, the white colonies obtained were randomly picked and cultured overnight at 37°C in 0.8 ml Luria broth (LB) medium supplemented with ampicillin (50 μg ml^−1^). The inserted DNAs were amplified by using M13-47 (5′-CGCCAGGGTTTTCCCAGTCACGAC-3′) and RV-M (5′-GAGCGGATAACAATTTCACACAGG-3′) primers targeting the flanking vector sequence, followed by agarose gel electrophoresis with ethidium bromide staining (Guan et al., 2013).

### Sequencing and phylogenetic analyses

Sequencing was carried out with an ABI 377 automated sequencer. After sequencing, reads were first trimmed for vector before subsequent analyses. Bellerophon was used to check for putative chimeric sequences (Huber et al., 2004). DNA sequences with more than 97% similarity were assembled into the same operational taxonomic units (OTUs) using FastGroup II (Yu et al., 2006), and one representative sequence was chosen from each OTU to compare with sequences in the GenBank Database using the BLASTX algorithm to identify nearest related ones (Altschul et al., 1997). Representative OTUs from clone libraries as well as reference sequences from GenBank were translated into corresponding amino acid sequences using EMBOSS Transeq tool (http://www.ebi.ac.uk/Tools/st/emboss_transeq/) with default parameter (Standard Genetic Code) and then aligned using Clustal Omega (Sievers et al., 2011). Phylogenetic trees were generated using MEGA5 software (Tamura et al., 2011). The topology of the trees was obtained by the Neighbor-Joining method (Saitou and Nei, 1987) with the Poisson correction method and 1000 bootstrap replicates were applied to estimate the support for the nodes in the tree.

### Nucleotide sequence accession numbers

Gene sequences data reported here are available in GenBank sequence database under the accession numbers KF111435–KF111455, KF111525–KF111548, KF111456–KF111492, KF111493–KF111501, and KF111502–KF111524 for *cbbM* gene, *cbbL gene*, *mcrA* gene, *fthfs* gene, and gene encoded by [Fe-Fe]-hydrogenase.

## Results

### Characterization of clone libraries

#### *cbbL* and *cbbM* genes

**Figure 2:**
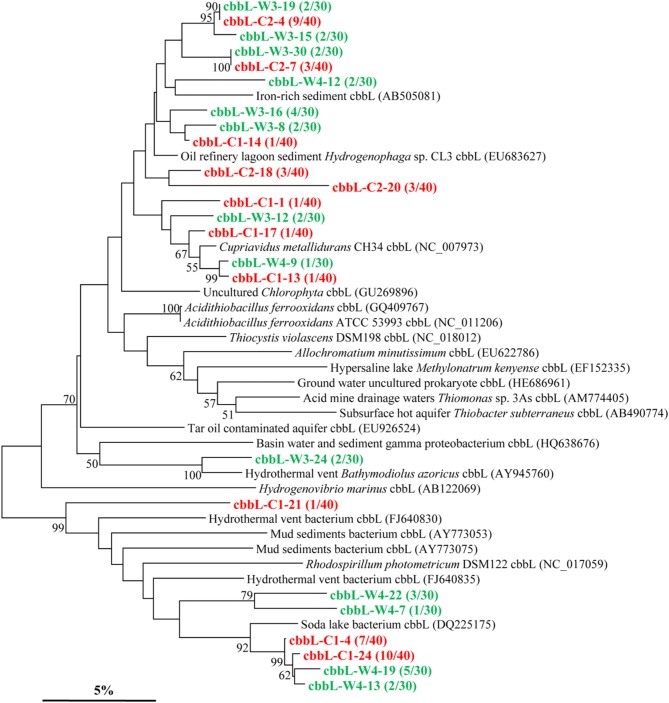
**Phylogenetic tree of the *cbbL* gene retrieved from the water samples (shown in colored) and closely related sequences from GenBank database**. Alignments to related sequences (shown with accession number) were performed with MEGA 5 software. The topology of the tree was obtained with the neighbor-joining method. Bootstrap values (*n* = 1000 replicates) greater than 50% are reported. Scale bar represents 5% amino acid substitution.

The *cbbL* gene types were positively detected in all two kinds of samples (Figure 2). The *cbbL* gene clone libraries from sample C and W resulted in 11 and 13 OTUs, respectively, and the PCR amplified sequences are spread over the entire tree. Phylogenetic analysis indicates that the *cbbL* gene sequences obtained are related to those of *Alpha*-, *Beta*-, and *Gamma-Proteobacteria. One* OTU (*cbbL*-C2-18) is closely related to *Hydrogenophaga* sp. CL3 affiliated to the family *Comamonadaceae* within *Beta-Proteobacteri*a (Garcia-Dominguez et al., 2008). The sequences of *cbbL*-C1-13, *cbbL*-C1-17, *and cbbL*-W4-9 all share high similarity with *Cupriavidus metallidurans* CH34 belonging to the family *Burkholderiaceae* within *Beta-Proteobacteria*. The sequence of *cbbL*-W3-24 shares high identity with endosymbiont of *Bathymodiolus azoricus* (Spiridonova et al., 2006), a member of *Gamma-Proteobacteria*. One OTU represented by *cbbL*-W4-12 shows highest identity with an uncultured bacterium from iron-rich environment (Kellermann et al., 2012).

**Figure 3:**
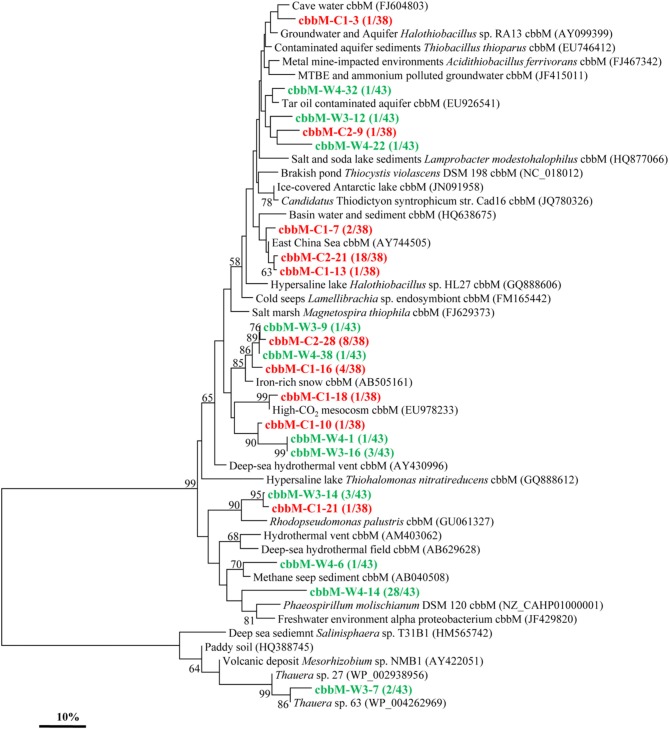
**Phylogenetic tree of the *cbbM* gene retrieved from the water samples (colored) and closely related sequences from GenBank database**. Alignments to related sequences (shown with accession number) were performed with MEGA 5 software. The topology of the tree was obtained with the neighbor-joining method. Bootstrap values (*n* = 1000 replicates) greater than 50% are reported. Scale bar represents 10% amino acid substitution.

Similarly, the *cbbM* gene types were also detected in these two samples and yielded 10 and 11 OTUs in C and W, respectively (Figure 3). The *cbbM* sequences detected are all very similar to those from organisms affiliated with members of *Alpha*-, *Beta*-, and *Gamma-Proteobacteria*. The sequence of *cbbM*-C1-3 is related to an uncultured bacterium from cave water of Romania (Chen et al., 2009). The OTUs represented by *cbbM*-W4-22, *cbbM*-W4-32, *cbbM*-W3-12, and *cbbM*-C2-9 are closely related to uncultured bacterium from tar contaminant aquifer and MTBE and ammonium polluted groundwater (Alfreider et al., 2012). Sequences represented by *cbbM*-C2-21*, cbbM*-C1-13, and *cbbM*-C1-7 all share similarities with those recovered from the East China Sea and basin water and sediment. Interestingly, these sequences are also closely related to *Halothiobacillus* spp., members of sulfur-oxidizing symbionts belonging to *Gamma-Proteobacteria*. Three OTUs (*cbbM*-C2-28, *cbbM*-W3-9, *cbbM*-W4-38, and *cbbM*-C1-16) are similar to an uncultured organism from iron-rich environmental samples (Kojima et al., 2009). Sequences represented by both *cbbM*-W3-14 and *cbbM*-C1-21 are closely related to *Rhodopseudomonas palustris*, a member of the order *Rhizobiales* within the *Alpha-Proteobacteria*. OTUs *cbbM*-W4-14 and *cbbM*-W4-6 representing 29 clones show highest similarities with *Phaeospirillum molischianum*, affiliated with the family *Rhodospirillaceae* within *Alpha-Proteobacteria* and with sequences from methane seep sediment. And *cbbM*-W3-7, which appeared to forms its own cluster, is related to *Thauera* spp. within the *Beta-Proteobacteria* and also to an uncultured bacterium from an environmental sample of paddy soil in China (Yuan et al., 2012).

#### *fthfs* genes

**Figure 4:**
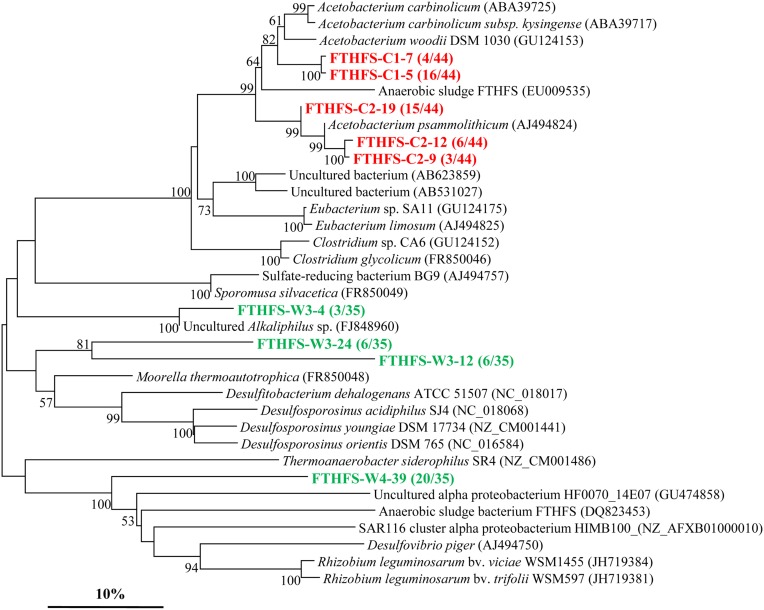
**Phylogenetic tree of the *fthfs* gene retrieved from the water samples (colored) and closely related sequences from GenBank database**. Alignments to related sequences (shown with accession number) were performed with MEGA 5 software. The topology of the tree was obtained with the neighbor-joining method. Bootstrap values (*n* = 1000 replicates) greater than 50% are reported. Scale bar represents 10% amino acid substitution.

The *fthfs* gene sequences were also detected in both samples. However, it showed a less abundant diversity as depicted in the phylogenetic tree (Figure 4) with the screened clones divided into 5 and 4 OTUs in sample C and W, respectively. Phylogenetic analysis shows that most of the *fthfs* gene sequences are related to members of the *Firmicutes*. Three OTUs (*FTHFS*-C2-9, *FTHFS*-C2-12, and *FTHFS*-C2-19) of sample C are all most similar to *Acetobacterium psammolithicum*, a member of the order *Clostridiales* within *Firmicutes* while 2 OTUs (*FTHFS*-C1-7 and *FTHFS*-C1-5) are obtained in sample C and sharing high similarities with *Firmicutes* members of the genus *Acetobacterium* (Xu et al., 2009). OTUs *FTHFS-W3-24* and *FTHFS*-W3-12 are related to sequences from genera *Moorella*, *Desulfitobacterium*, and *Desulfosporosinus*, also members of the *Firmicutes*. *FTHFS*-W3-4 is similar to uncultured *Alkaliphilus* sp. from anaerobic wastewater of Mesa Northwest Wastewater Reclamation Plant (Parameswaran et al., 2010).

#### [FeFe]-hydrogenase-encoding gene

**Figure 5:**
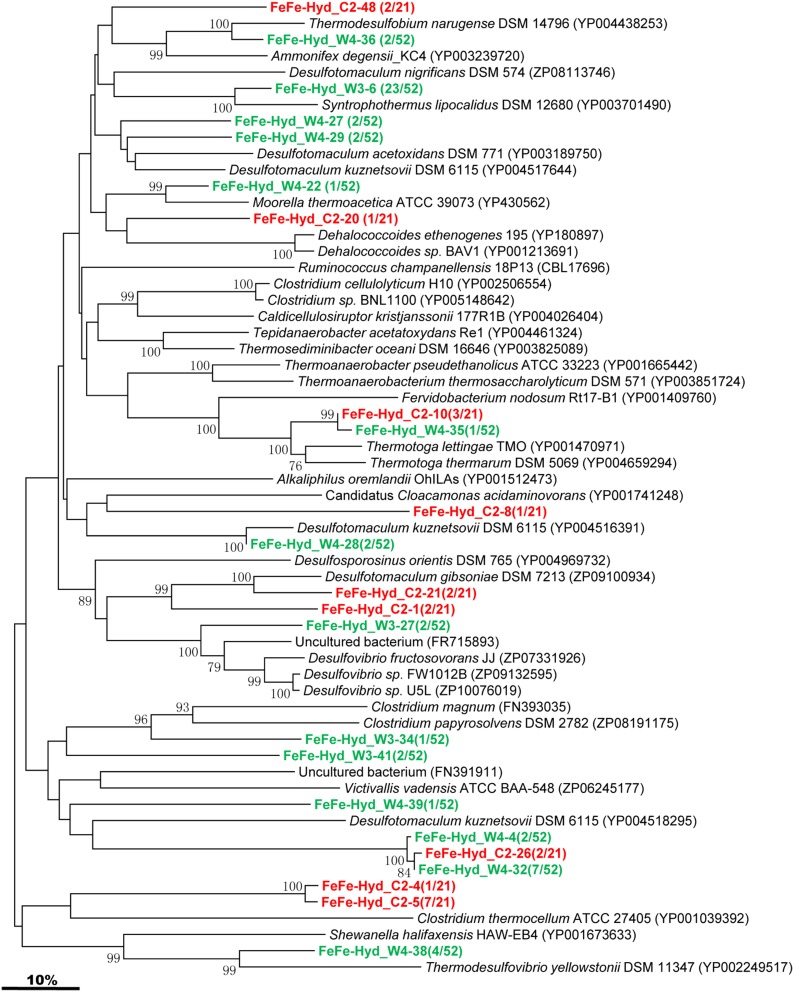
**Phylogenetic tree of the *[FeFe]-Hydrogenase* gene retrieved from the water samples (colored) and closely related sequences from GenBank database**. Alignments to related sequences (shown with accession number) were performed with MEGA 5 software. The topology of the tree was obtained with the neighbor-joining method. Bootstrap values (*n* = 1000 replicates) greater than 50% are reported. Scale bar represents 10% amino acid substitution.

The [FeFe]-hydrogenase-encoding gene was detected in both C and W samples, and phylogenetic analysis of the sequenced clones were assembled into 9 and 14 OTUs, respectively (Figure 5). The majority of the gene sequences obtained from the two samples cluster with sequences related to *Firmicutes*. One OTU represented by FeFe-Hyd_W3-6 shares similarity with *Syntrophothermus lipocalidus* of the *Firmicutes*. FeFe-Hyd*_W4-38* is either related to *Shewanella halifaxensis* HAW-EB4 within the *Gamma-Proteobacteria* or to *Thermodesulfovibrio yellowstonii* within the *Nitrospira* (Figure 5). FeFe-Hyd_W4-36 is related to *Thermodesulfobium narugense* belonging to the family *Thermodesulfobiaceae* within the *Firmicutes*. FeFe-Hyd_W4-22 shares high identity with *Moorella/ thermoacetica* affiliated to the family *Thermoanaerobacteraceae* of *Firmicutes*. FeFe-Hyd_W4-4, FeFe-Hyd_C2-26, and FeFe-Hyd_W4-32 are all related to *Desulfotomaculum kuznetsovii*, a member of the order *Clostridiales* within *Firmicutes*. FeFe-Hyd_C2-10 and FeFe-Hyd_W4-35 are both similar to *Thermotoga lettingae* TMO affiliated with the family *Thermotogaceae*.

#### *mcrA* genes

**Figure 6:**
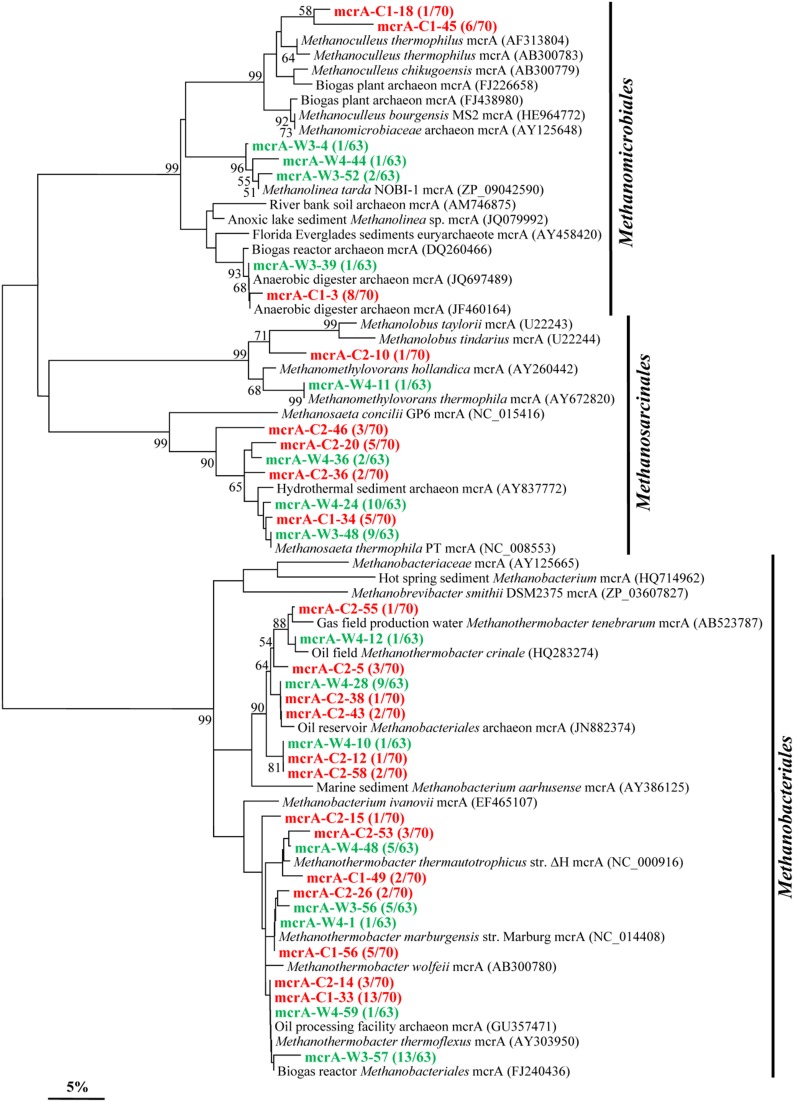
**Phylogenetic tree of the *mcrA* gene retrieved from the water samples (colored) and closely related sequences from GenBank database**. Alignments to related sequences (shown with accession number) were performed with MEGA 5 software. The topology of the tree was obtained with the neighbor-joining method. Bootstrap values (*n* = 1000 replicates) greater than 50% are reported. Scale bar represents 5% amino acid substitution.

By using *mcrA*-targeted specific PCR primers set, 21 and 16 OTUs (37 overall) were obtained in samples C and W, respectively (Figure 6). Phylogenetic analysis shows that 21 OTUs (13 in C and 8 in W) are all closely related to sequences from members affiliated to the *Methanobacteriales*, an order known to harbor mostly CO_2_-reducing methanogens. A total of 7 OTUs (3 in C and 4 in W) shared high identities with *mcrA* sequences from the *Methanomicrobiales*. And 9 OTUs (5 in C and 4 in W) are closely related to sequences affiliated to methylotrophic and acetoclastic methanogens within the order *Methanosarcinales*.

#### Characterization of functional microbial communities

**Figure 7:**
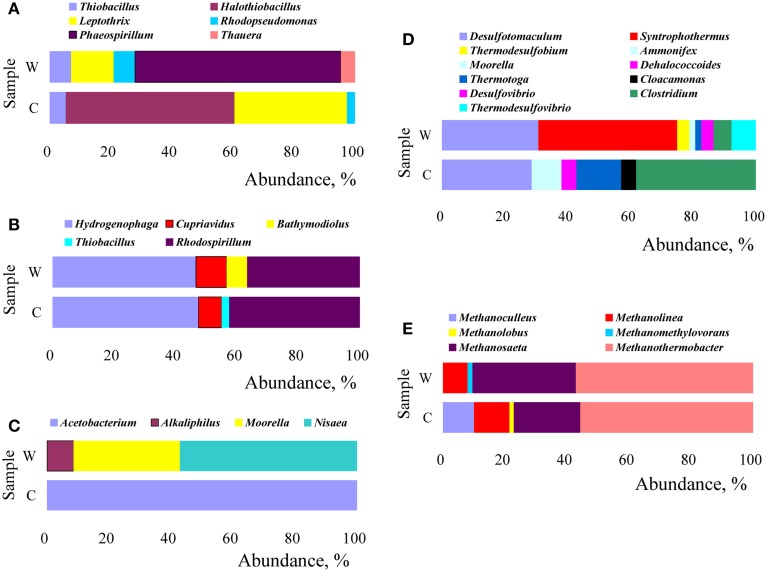
**Relative abundance of functional microbes (at the genus level) with respect to the sequences retrieved by functional marker genes of *cbbM* (A), *cbbL* (B), *fthfs* (C), *FeFe*-hydrogenase(D), and *mcrA* (E)**.

Changes in microbial structure were analyzed by their relative abundance calculated from the number of clones and the results were showed in Figure 7. The community structure of microorganisms with most similarity to the retrieved amino acid sequences of *cbbM* gene was distinct in W and C samples (Figure 7A). The genera *Phaeospirillum* (67.4%), *Leptothrix* (14.0%), *Rhodopseudomonas* (7.0%), and *Thiobacillus* (7.0%) were dominant in W sample, whereas, *Halothiobacillus* (55.3%) and *Leptothrix* (36.8%) were dominant in C sample. In the *cbbL* clones libraries (Figure 7B), the genera *Rhodospirillum* 36.7% and 42.5%, *Hydrogenophaga* 46.7% and 47.5%, *Cupriavidus* 10.0% and 7.5% were dominant in W and C sample, respectively. As for the composition of *fthfs* communities (Figure 7C), in W sample, the community was mainly composed by microorganisms related to genera *Nisaea* (57.1%), *Moorella* (34.3%), and *Alkaliphilus* (8.6%), however, only by microorganisms related to genus *Acetobacterium* (100%) in C sample. It can be seen from Figure 7D that C sample was dominantly composed by microbes related to members of genera *Clostridium* (38.1%), *Desulfotomaculum* (28.6%), and *Thermotoga* (14.3%), while the W sample by *Syntrophothermus* (44.2%) and *Desulfotomaculum* (30.8%). Meanwhile, those related to *Ammonifex*, *Dehalococcoides*, and *Cloacamonas* all rose in relative abundance from undetectable in W sample to 4.8% in C sample. The methanogen community was demonstrated in Figure 7E. As shown in Figure 7E, thermophilic *Methanothermobacter* (55.7% and 57.1% in C and W sample, respectively), *Methanolinea* (11.4% and 7.9% in C and W sample, respectively), and *Methanosaeta* (21.4% and 33.3% in C and W sample, respectively) were the predominant methanogens. *Methanoculleus* (10.0%) were only detected in the C sample.

## Discussion

### Occurrence of microorganisms associated with CO_2_ sequestration in oil reservoirs

The microbial community structure in production water samples in Daqing oilfield of China was analyzed by means of a suite of functional genes as biomarkers. Our results indicate that members of the *Proteobacteria* (*Halothiobacillus*, *Leptothrix*, *Hydrogenophaga*, and *Rhodospirillum*) were the predominant ones with the ability of fixation of CO_2_ in *in situ* oil reservoirs. It has been reported that the CBB cycle for CO_2_ fixation operates in *Proteobacteria* belonging to the *alpha*-, *beta*-, and *gamma*-subgroups, and some members of the *Firmicutes* (Zakharchuk et al., 2003; Caldwell et al., 2007). In addition, the acetogens belonging to *Clostridiaceae* within *Firmicutes* can use the reductive acetyl-CoA pathway not only for CO_2_ fixation but also for the production of acetic acid, which is substrate for methanogenesis. Other major bacterial sequences in the clone libraries of sample W are related to those of *Hydrogenophilaceae*, and similar microorganisms were reported to use the rTCA cycle for autotrophic CO_2_ fixation (Schauder et al., 1987; Thauer et al., 1989). For the archaeal *mcrA* gene clone libraries, the predominance of the genus *Methanothermobacter* belonging to hydrogenotrophic methanogens is notable.

The majority of *cbbL* gene types obtained were very similar to the microorganisms belonging to *Alpha*-, *Beta*-, and *Gamma-Proteobacteria*. And some members of these phyla have been reported in previous studies, but of which *Hydrogenophaga* sp. and *Cupriavidus* sp. were rarely documented (Alfreider et al., 2003). The *cbbM* gene types detected are also related to those of *Alpha*-, *Beta*-, and *Gamma-Proteobacteria*, and this is consistent with the research results of Hugler et al. (2010). All above data suggest that microorganisms within *Proteobacteria* mainly use the CBB cycle for CO_2_ fixation in the oil reservoirs studied.

Acetogenic bacteria are among the most phylogenetically diverse bacterial functional groups. To date, approximately hundreds of homoacetogenic species have been identified and phylogenetically classified into 21 different genera. The *fthfs* gene sequences obtained from CO_2_-flooded fraction of the reservoir shared high similarities with those from members of the *Firmicutes* with most of the sequences related to the order *Clostridiales*, deducing that microorganisms affiliated with *Firmicutes* inhabiting the herein investigated oil reservoirs have the ability to fix CO_2_ as well as convert CO_2_ into acetic acid via the acetyl-CoA pathway.

H_2_ is necessary to *in situ* CH_4_ production by hydrogenotrophic methanogens in oil reservoirs. In the present study, we found that sequences from microorganisms similar with those from the *Firmicutes*, *Gamma-Proteobacteria*, and *Thermotogae* were the most encountered in clone libraries established for [FeFe]-hydrogenase-encoding gene, and these results are consistent with those of Schmidt et al. (2010), who found that members of the order *Clostridiales* and *Thermoanaerobacter* sp. were likewise all capable of fermentative production of H_2_ (Schmidt et al., 2010).

Methanogenesis is the terminal step of organic compound degradation and plays a major role in the global carbon cycle (Garrity and Holt, 2001; Liu and Whitman, 2008). The most important precursors for methane production during anaerobic digestion of organic matter are H_2_-CO_2_ and acetate, which are converted into methane by hydrogenotrophic and aceticlastic methanogens (Mayumi et al., 2011), respectively. Interestingly, it is proposed that syntrophic acetate oxidation coupled to hydrogenotrophic methanogenesis is an alternative methanogenic pathway in petroleum reservoirs (Mayumi et al., 2011). Analysis based on the *mcrA* gene types indicates 12 OTUs detected share high identity with those of the genus *Methanothermobacter*.

To the best of our knowledge, the collection of functional genes described in the present work has not yet been investigated in oil reservoir systems, although some of them have been reported in geothermal environments. The detection of CO_2_ fixation genes as well as hydrogenases-encoding and *fthfs* genes in production fluids of high temperature oil reservoirs provides new insights on the diversity and composition of microorganisms involved in the microbial fixation of CO_2_ and its subsequent conversion to methane.

### Impact of CO_2_ injection on specific microbial communities with respect to microbial fixation and bioconversion of CO_2_

Microbial fixation and conversion of CO_2_ into methane in oil reservoir by indigenous microorganisms is one of the most promising solutions to the mitigation of CO_2_ emission. We explored the potential for autotrophic CO_2_ fixation and bioconversion with microbial communities in oil reservoir by detection of relative functional biomarker genes such as CO_2_ fixation (*cbbM*, *cbbL*), acetogenesis (*fthfs*), hydrogen formation ([FeFe]-hydrogenase-encoding gene), and methanogenesis (*mcrA*). Microbial fixation and conversion of CO_2_ are usually implemented by chemolithoautotrophic microorganisms, which usually obtain their energy through the oxidation of inorganic compounds and utilization of CO_2_ as their sole source of carbon. Thus, the CO_2_ injected as well as the subsequent changes in pH and other geochemical parameters induced by CO_2_ have an influence on the metabolism of the both heterotrophic and lithoautotrophic microorganisms (Ramos, 2003). Therefore, injection of CO_2_ may cause some changes in microbial populations as well as their activities, and it is important to characterize these changes with respect to CO_2_ fixation and bioconversion to methane.

Methanogens use molecular hydrogen (H_2_) anaerobically by transferring electrons from H_2_ to CO_2_ to form methane. As demonstrated in Figure 7E, Thermophilic *Methanothermobacter*, *Methanolinea*, and *Methanosaeta* were predominant methanogens both in W and C samples. With comparison to W sample, the promotion in relative abundance of *Methanolinea* (from 7.9 to 11.4%) and *Methanoculleus* (from undetectable to 10.0%) as well as the reduction in relative abundance of *Methanosaeta* (from 33.3 to 21.4%) were observed, which implied that the injected CO_2_ influenced negatively on *Methanosaeta* but positively on *Methanoculleus* and *Methanolinea*. Considering that *Methanothermobacter*, *Methanolinea*, and *Methanoculleus* are known to be hydrogenotrophic methanogens, *Methanosaeta* to aceticlastic methanogens, and *Methanomethylovorans* to methylotrophic methanogens, it is reasonable to conclude that injection of CO_2_ either increase or maintain the relative abundance of hydrogentropic methanogens, but it decreases that of aceticlastic methanogens and methylotrophic methanogens.

More interestingly, *Methanoculleus* was detected only in C sample. The genus has been found in different habitats including oil reservoir (Berdugo-Clavijo and Gieg, 2014), deep marine sediments (Mikucki et al., 2003), and swine manure storage tank (Barret et al., 2012, 2013). The occurrence of this genus in C sample implies that it may be related to CO_2_ injection driven high acetate concentration. This assumption is consistent with the fact that *Methanoculleus* spp. consume acetate while carrying out hydrogenotrophic methanogenesis and the growth of some *Methanoculleus* members requires acetate even though they do not convert it to methane (Mikucki et al., 2003; Barret et al., 2013, 2015). Also, Berdugo-Clavijo and Gieg found that the relative abundance of *Methanoculleus* decreased substantially with acetate (Berdugo-Clavijo and Gieg, 2014). In this study, the C-water is highly enriched in acetate relative to W, which one might normally assume favors aceticlastic methanogens. Based on the known properties of *Methanoculleus* spp., it seems that the acetate is favoring acetate assimilating methanogens.

Ribulose 1, 5-bisphosphate carboxylase (Rubisco, specifically, *cbbL*, *cbbM*) are usually used as a biomarker for the CBB CO_2_ fixation pathway (Campbell and Cary, 2004). Specifically, in subsurface environments, CO_2_ fixation is usually conducted by chemolithotrophs through the CBB pathway (Kellermann et al., 2012). As Figure 7A showed, the most dominant genus *Phaeospirillum*(67.4%) in W sample was not detected in C sample and the abundance of *Thiobacillus* and *Rhodopseudomonas* in C sample decreased notably while compared to W sample. In addition, the *Halothiobacillus* (undetected in W sample) appeared to be the most prevalent in C sample. Also, the relative percentage of *Leptothrix* in C sample increased compared to that in W sample. In the *cbbL* clones libraries, the abundance of *Rhodospirillum* increased in abundance from 36.7% in W sample to 42.5% in C sample, members of the genus *Hydrogenophaga* increased in abundance slightly in C sample compared to that in W sample, while those affiliated to genus *Cupriavidus* decraed from 10.0% in W sample to 6.5% in C sample (shown in Figure 7B). Alfreider et al. (2003) also detected *Hydrogenophaga*, *Thiobacillus*, and others related *cbb* sequences in a contaminated aquifer. The abundance and diversity of the detected *cbb* genes hint at a significant potential for CO_2_ fixation via the Calvin cycle within oil reservoir microbial communities.

Most acetogens are obligate anaerobic bacteria that use the reductive acetyl-CoA pathway as their main mechanism for energy conservation and for synthesis of acetyl-CoA and cell carbon from CO_2_. Formyltetrahydrofolate synthetase (*fthfs*) is used to detect acetogenic, fermentative bacteria (Leaphart and Lovell, 2001). In the present work, notable changes were observed in the composition of *fthfs* communities (Figure 7C). The community dominated by microorganisms related to genera *Nisaea*, *Moorella*, and *Alkaliphilus* in W sample was changed completely to be dominated only by microorganisms related to genus *Acetobacterium* in C sample. The mechanism for the change of *Alkaliphilus* from dominance in sample W to undetectable in sample C is not very clear. Generally, this genus is known to be extremely alkaliphilic and thus would not be prone to survive in the acidic conditions caused by the injection of CO_2_. Although the ability of acetate production on CO_2_+H_2_ by *Acetobacterium woodii* and *Moorella* were systematically studied (Ragsdale and Pierce, 2008; Demler and Weuster-Botz, 2011), surprisingly, *Moorella*-like microorganisms were not detected in C sample. This observation implies that *Acetobacterium*-like microbes are probably more suitable for acetogenesis in CO_2_-injected oil reservoirs.

Hydrogen is an alternative energy source for autotrophic microbes in a variety of subsurface environments. When hydrogen and carbon dioxide are present, development of autotrophic microorganisms would be possible. For example, methanogens and acetogens may produce organic matter from hydrogen by means of respiring carbon dioxide. As it can be seen from our study (Figure 7D), the composition of [FeFe]-hydrogenase-encoding gene clones libraries at the genus level shows interesting differences in relative abundance between W and C samples. The microbes related to *Syntrophothermus* predominated in W sample (44.2%) disappeared in C sample, and *Desulfotomaculum* decreased from 30.8% in W sample to 28.6% in C sample. The relative abundance of members of genera *Clostridium*, *Thermotoga*, and *Ammonifex* increased from 5.8%, 1.9% and undetectable (0.0%) in W sample to 38.1%, 14.3% and 9.5% in C sample, respectively. Interestingly, all the three sulfate reducing bacteria were influenced very markedly as either decreased in relative abundance (*Desulfotomaculum*) or became undetectable in C sample (*Thermodesulfobium* and *Thermodesulfovibrio*). Meanwhile, those related to *Dehalococcoides* and *Cloacamonas* all rose in relative abundance from undetectable in W sample to 4.8% in C sample. Morozova et al. (2010) also showed that CO_2_ injection caused a decrease in the diversity of microorganisms and revealed temporal out-competition of sulfate-reducing bacteria by methanogenic bacteria. Morozova's experiments showed that after CO_2_ injection the SRB population declined until it was no longer detected while the archaeal population increased, which indicates that archaea may be able to adapt more readily to the more acidic conditions after CO_2_ injection. Our results reached the same conclusion. But, Morozova found that after a 5 month period of exposure to CO_2_, the SRB population returned in numbers greater than that prior to CO_2_ injection. This phenomenon was not observed in our study at present. The reason for this was not quite clear although it was assumed to be resulted partly from the water–gas alternative injection and long-term exposure of CO_2_ (about 5 years) in our study which were quite different from that in Morozova's experiments.

We found great differences in relative abundance among all the five functional gene clone libraries established from W and C samples, as showed in Figure 7. This phenomenon of previously undetectable and/or rare members of microbial communities becoming dominant after exposure to CO_2_ has been reported previously (Gulliver and Gregory, 2011). Microorganisms with increasing abundance implies that they may be better withstanding or adapting to exposure to CO_2_ and subsequent changes in physical and biochemical conditions resulted by CO_2_ injection.

Analysis of functional genes shows that microbial communities were strongly influenced and the diversity reduced by CO_2_ injection. For example, there were eight different genera in W sample whereas only six were retrieved from C sample for [FeFe]-hydrogenase-encoding gene library. Also, for *fthfs* library, three different genera were detected in W sample but only one was found in C sample. Our data agree with Gulliver and Gregory (2011) which showed that different families of bacteria presided with variation in CO_2_ partial pressure. Knowledge of surviving and thriving microbial populations may help in better understanding of the fate of CO_2_ following injection and to make better strategy for use of microorganisms in subsurface environments for improving the efficiency of injection and microbial fixation of CO_2_, and hence ensuring the security for long-term CO_2_ storage in subsurface petroleum reservoirs.

Primers used for *mcr*A amplification are divided into different groups: MCR, ME, ML, and these primers are able to amplify most methanogens. It has been reported that the ME-related primers are also able to amplify anaerobic methane-oxidizing archaea (ANME) (Narihiro and Sekiguchi, 2011). The primers used for *mcr*A amplification to target the methanogenic communities in the samples investigated in the present study were described by Luton et al. (2002) which belonged to the ML group. To the best of our knowledge, the ML group ability to amplify ANME's remains to be demonstrated.

Due to the fact that the CO_2_ injected had been produced about 1 year before the collection of these samples when the ratio of gas (CO_2_) to oil was between 22.8 and 145 m^3^/m^3^ in production wells, the changes in the relative abundance of five genes relevant to CO_2_ utilization and methane production by microorganisms can be considered mainly attributed to CO_2_ injection. The small size of the clone library and the number of clones sequenced would influence, to some extent, on the analysis of microorganisms with low frequency. Nevertheless, the major functional microorganisms and their changes in relative abundance can still be recognized, even with certain biases, as demonstrated in the present study. The analysis of the changes in microbial community may be influenced by the following factors: (1) The samples were all collected from the sampling valve located at the wellhead of production well and hence, these samples may contain microbes from oil reservoir as well as that survived in oil tubes between the well bottoms to the sampling valve; (2) The sampling water may be produced both from oil-bearing layers or sub-layers with CO_2_ production (CO_2_-impacted water) and that with no CO_2_ production even they received CO_2_(non CO_2_-impacted water); (3) The retention time of CO_2_ in oil reservoir is relatively short, i.e., while CO_2_ was injected through injection wells into target oil reservoir, part of them would be produced afterwards from the production well about 250–300m away from the injector; (4) CO_2_ was injected into the target oil reservoir with water-CO_2_ alternative injection manner.

For a more accurate characterization of microbial community and their changes caused by CO_2_ injection in oil reservoir, the collection of produced water from only the CO_2_-impacted zones, the qualitative and quantitative analysis of microbial community, the physiochemical changes of subsurface water such as pH, volatile acids over time, as well as the analysis of the origin of volatile acids (by isotopic analysis) and etc. are very important.

### Methane formation potential from injected CO_2_ in oil reservoirs

Bioconversion of CO_2_ into CH_4_ *in situ* oil reservoirs by indigenous methanogens is an area of active research and development. Hydrogenotrophic methanogens need not only CO_2_ but also H_2_ to produce CH_4_; therefore, H_2_ should be supplied to them in reservoirs for this process. It has been reported that there are several kinds of microorganisms capable of producing H_2_ by degrading crude oil in reservoir environments. The potential of the microbial conversion of CO_2_ into CH_4_ by enrichment culture experiments using microorganisms indigenous to oil reservoirs has been studied (Sugai et al., 2012). Different from that mentioned above, we evaluated the potential of this process from the viewpoint of functional genes. In our study, both the functional genes of H_2_-producing and CH_4_-producing were detected in the CO_2_-flooding oil reservoirs, and the water-flooding oil reservoirs as well. Furthermore, some H_2_-producing microorganisms (e.g., *Clostridium* and *Thermotoga*) and hydrogenotrophic methanogens such as *Methanothermobacter* and *Methanolinea* as well as *Methanoculleus* remained or evolving to be predominant after long term exposure to CO_2_ in CO_2_-flooding area compared to that in water-flooding area. Meanwhile, these H_2_-producing bacteria and hydrogenotrophic methanogens were both identified in the 16S rRNA genes cloning libraries (data not shown in this paper). It is assumed that these hydrogenotrophic methanogens live in symbiosis with hydrogen-producing bacteria and convert CO_2_ into CH_4_ in oil reservoirs. These results indicate that indigenous microbial conversion process of CO_2_ into CH_4_ has high potential.

The detection of CO_2_ fixation potential is alternative evidence to autotrophic activity *in situ* oil reservoirs. Therefore, attentions should be further paid on the evaluation of the activities of those microorganisms in subsurface ecosystems with the potential of microbial fixation of CO_2_ and its subsequent bioconversion into methane. Once those microorganisms are activated by means of nutrient injection and etc., taking into consideration of the tremendous capacity of CO_2_ sequestration in oil and gas reservoir (totally about 9 × 10^11^ tons in the world), it seems more reasonable to believe that the *in situ* fixation and reclamation of CO_2_ sequestrated in oil reservoir will play an notable role in mitigating atmospheric CO_2_ building up as well as energy shortage.

## Conclusions

Analysis of a suite of functional genes shows that a diverse microbial community with potential for fixation and conversion of CO_2_ into methane inhabits oil reservoir. Microorganisms affiliated with members of the genera *Methanothemobacter* (hydrogenotrophic CO_2_-reducing methanogens), *Acetobacterium* and *Halothiobacillus* as well as hydrogen producers (*Firmicutes*) seem to be more adaptable to CO_2_ injection and present the potential for microbial fixation and bioconversion of CO_2_ into methane in subsurface oil reservoirs. Due to the limitation of clone numbers and the co-production nature of CO_2_-impacted and non-impacted water in the C sampling well, the impact of CO_2_ injection on microbial community may be not fully characterized and presented in this study. Even so, the present results showing the response, to some extent, of microbial community on the CO_2_ injection are of some help in predicting the fate of CO_2_ following injection and making better strategies for use of microorganisms in subsurface environments for microbial CO_2_ fixation and bioconversion of CO_2_ into sustainable energy in subsurface oil reservoirs.

## Author contributions

This study was designed by JL and BM. XS and GY performed all the laboratory experiments. SM analyzed the functional genes data and constructed the phylogenetic trees of these functional genes. JG provided valuable suggestions in the design of the experiments and the preparation of the manuscript. The manuscript was written by JL, assisted by all co-authors. All authors reviewed the final manuscript.

### Conflict of interest statement

The authors declare that the research was conducted in the absence of any commercial or financial relationships that could be construed as a potential conflict of interest.
